# Supplementary material for: MetaRibo-Seq measures translation in microbiomes
Source: Nat Commun. 2020 Jun 29;11:3268. doi: 10.1038/s41467-020-17081-z (PMC7324362; doi:10.1038/s41467-020-17081-z)
Supplement: Supplementary file 10 — Supplementary Data 7 [file 41467_2020_17081_MOESM10_ESM.zip › File2/Confidence_VeryHigh_Taxonomy/250140_out.krona.html]

Javascript must be enabled to view this page.

members
magnitude
magnitudeUnassigned
count
unassigned
taxon
rank

250140\_out

10

superkingdom
9
10239

10841
9
family


SRS011134\_contig\_number\_contig-100\_894.283170SRS020233\_contig\_number\_contig-100\_675.337766SRS054956\_contig\_number\_contig-100\_3396.78986
3
species
2202644

686565
6
subfamily


SRS049959\_contig\_number\_49308SRS050925\_contig\_number\_27390SRS1041033\_contig\_number\_21258SRS1041133\_contig\_number\_1489SRS104400\_contig\_number\_51213SRS147557\_contig\_number\_contig-100\_1297.187771
6
species
1758150

2
superkingdom
1

phylum
1
1239

1
class
186801


SRS013476\_contig\_number\_contig-100\_2350.2350
186802
order
1
